# Some Rare Cases in Obstetric Practice, with Special Reference to the Treatment

**Published:** 1895-05

**Authors:** C. H. Richardson

**Affiliations:** Montezuma, Ga.


### SOME RARE CASES IN OBSTETRIC PRACTICE, WITH SPECIAL REFERENCE TO THE TREATMENT.

By C. H. RICHARDSON, MONTEZUMA, GA.

It is not that I wish to present any new idea, or to unfold any new scientific investigation, nor is it to display cases successfully treated and to leave off those that have gone to the Great Beyond, that I use this subject and bring before you cases which I recall from my note book of years, but that we might gain from each other lessons of experience some of the rare cases that will sooner or later fall to the lot of us all.

From an extensive obstetric practice of thirteen years, I have selected a few rare and grave cases, which I shall arrange in the order of their occurrence:

First.—"*Cæsarean Section.*"—Mollie M., a negress, multipara, living in a filthy little negro hut, eight miles in the country, had been in labor forty-eight hours under the supervision of a midwife, when I arrived on December 15, 1885. Upon an examination I found tetanic contraction of uterus. So strong were these contractions, that it was impossible to make a positive diagnosis of the presentation even under an anæsthetic, also impossible to turn under complete anæsthesia. Myself and my colleague, Dr. R. O. Engram, who was present with me, decided nothing could be done except to perform Cæsarean section. Preparing the patient and instruments as thoroughly antiseptic as we could under the circumstances, I made the usual incision through the linea alba,. peritoneum and uterus, and extracted a living child; a red spot on left cheek bone of the infant indicated the presentation. The uterus, deep fascia and skin were united by interrupted carbolized ligatures. Reaction was brought about by free stimulation and strychnine. Patient was treated antiseptically, but from the fact of lying almost in a bed of germs, in this miserable hut, died on Sunday of peritonitis. The child is living to-day.

Second.—"*An Elongated Cervix Mistaken for Placenta Prævia.*"—Blanche B., a negress, multipara. Was hastily summoned on December 15, 1892, with the statement that the midwife, an intelligent old white lady, said that the placenta had come down first. On my arrival, in answer to my questions, the patient stated that she was taken in labor on the previous morning (24 hours prior to this time) two miles from her home, and in order to reach her place of abode, and prepare her bed before the noted event, she ran rapidly across cotton fields in order to get home sooner. Upon examination I soon saw that there was no hemorrhage, and that there had been none, but on pushing my investigation, my finger came in contact with a large, thick, fleshy substance as large as my hand, which really felt like a placenta, but on tracing it up further, soon found out that it was the product of an old lacerated cervix. The strangest part of the proceedings to me was how this cervix or portion of it, became so large and so elongated as to cause obstruction, and prevent the termination of labor.

It possibly may have been due to her running across the fields, and the rough handling that she received afterwards. I found it impossible to push this mass up above the fœtal head and apply the forceps, so with one hand I pulled it down as far as possible into the vagina on the left side, applied the forceps and readily extracted a living child, with the exclamation of "God bless you" from the patient.

Third.—"*Shoulder Presentation.*"—This case was one of a gypsy woman encamped a few miles from town in a pine forest. Was called on January 30, 1892, but being engaged at the time, told the messenger if she were not delivered by morning I would go out. I was summoned quite early the next morning. When I arrived I found a gypsy woman of middle age lying on a cot under a tent, situated in a lonely pine forest, with no one present but her husband and an old ignorant negro midwife. Upon inquiring of the old midwife what was the trouble, she replied, "I dunno, sir; I dun gib dat creature ebbery ding from piping her to gunpowder tea, and I aint made her break her holt yit."

Upon examination I found that I had a shoulder and arm presentation to contend with, but on account of the tetanic contractions of the uterus, it was impossible to get the hand in the uterus to turn even under anæsthesia, and the arm of the child as in the way, preventing turning by podalic version. As the child was already dead, I pulled the arm down and disarticulated it at the shoulder-joint, then with difficulty running my hand up the uterus, I grasped a foot and performed version in this way. I delivered the child with difficulty as the contracted uterus would grasp the child's head after the body was delivered. Strange to say, under antiseptic treatment she got on without any trouble and started on her journey in about ten days.

Fourth.—"*Partial Separation of the Placenta.*"—Mrs. S., primipara, white. On the afternoon of October 20th, while walking down the doorsteps, stumbled and fell to the bottom. She only received apparently a slight shock, and very little inconvenience from the fall, but in a few hours she noted that there was some oozing of blood from her, accompanied by a slight uterine contraction. About twelve o'clock I was summoned to her. As labor had commenced and the hemorrhage was not great, I decided to wait awhile on nature. Being of a naturally nervous nature (her sister a few months before having had a serious time), she was exceedingly more so from the fact that she knew she was having some hemorrhage. It was my idea to make the head come down as far as possible, so as to form a wedge or constriction to placenta, thereby preventing so much hemorrhage; so as soon as the os was fully dilated, I broke the bag of waters. I then waited several hours on nature, but as I saw my patient was gradually losing strength, probably from internal hemorrhage, I applied the forceps and delivered the child, which was asphyxiated. Probably the death of the infant was caused from hemorrhage of the placenta, cutting off its circulation, or strangulation by blood getting into its mouth. As soon as the child was delivered there was an alarming hemorrhage, which almost completely exsanguinated my patient and would have proven fatal in a few moments had I not grasped the uterus with my hand and brought on uterine contractions. She had a long and tedious recovery. She has since borne two children, both breech presentations. One was so completely asphyxiated that all the old women present said it would be wasting time to attempt to resuscitate it; but, after laboring with her for over sixty minutes, life began to be manifested and it is a living child to-day.

Fifth.—"*Albuminuria.*"—Mrs. T., white, primipara. Was called one evening to see her for a severe headache as she had not yet expected to be confined, and up to this time had enjoyed excellent health. No œdema about any part of the body. On my arrival she remarked that she was sorry that he had sent for me, as she then felt better; but in a few minutes an intense headache began, and as she expressed it, of a boring nature. She remarked that she could hardly see me. I soon surmised what I had to contend with, but little did I dream that it would be so rapidly fatal. Told her that I would give her a dose of phenacetine to relieve her head. I gave her five grains and she seemed to get easy, but I soon noticed how strangely she was breathing; went to her and found her completely unconscious and could not be aroused. I could not get anything down her, so tried to bring on free cartharsis by dry calomel on the tongue and action upon the skin by mustard footbaths and pilocarpine. Being a strong healthy woman with a strong pulse, I decided she would be a good subject to bleed, so took nearly two pints of blood in all from left vein. I drew off some urine from the bladder and upon examination it consisted of nearly forty per cent. of albumen. There was at no time any indication of labor, neither had there been any unfavorable symptoms before I was called. I did not attempt to bring on labor. It has been a question with me ever since whether I should have done so. In little over six hours from the time of my arrival my patient was dead. I keenly felt the futility of my remedies in this grave case, and under similar circumstances I don't know that I could have done any better. I more keenly felt it from the fact that in an obstetric practice of over two hundred white cases, this was the first one that I had lost.

Sixth.—"*Albuminuria, With Convulsions.*"—Was hastily called on June 9th to see Mrs. A., multipara, near term, who sent for me during the absence of her regular physician. As I reached her door they requested me to see her hurriedly, as she had another convulsion, having had one half an hour previously, when they first sent for me. As I walked in she was in the midst of a hard convulsion, which seemed to be in every muscle of her body, and lasted several minutes. I gave her at once one-eighth of a grain of morphia and $\frac{1}{120}$ grain of atropia, hypodermically, and as soon as she regained consciousness I gave her eight drops of Norwood's tincture every two hours until I got the pulse down to eighty per minute, then I continued with 5 drops every three hours till the danger had passed of a return of the convulsion. In order to set the kidneys to acting and remove the effete materials from the blood, I also put her on two grains of calomel and one grain of powdered digitalis every two hours till there was free catharsis. She never had another convulsion from this time (6 A. M.) till 5 P. M. that afternoon, when she again had another convulsion, but soon after this she had several movements of the bowels, due to the mercury, and the convulsions never returned after this. Remaining with her all night, by next morning she was doing so nicely that I placed her on acetate of potash and tincture digitalis, to be taken four times daily, with request to call me if needed.

She went on for two weeks to regular time and was confined without any trouble in the least.

